# Placental adaptations supporting fetal growth during normal and adverse gestational environments

**DOI:** 10.1113/EP090442

**Published:** 2022-12-09

**Authors:** Amanda Nancy Sferruzzi‐Perri, Jorge Lopez‐Tello, Esteban Salazar‐Petres

**Affiliations:** ^1^ Centre for Trophoblast Research, Department of Physiology, Development and Neuroscience University of Cambridge Cambridge UK; ^2^ Facultad de Ciencias Departamento de Ciencias Básicas, Universidad Santo Tomás Valdivia Chile

**Keywords:** ageing, fetus, hypoxia, malnutrition, mitochondria, nutrient, obesity, placenta, pregnancy, sex differences

## Abstract

The placenta is vital for mammalian development and a key determinant of life‐long health. It is the interface between the mother and fetus and is responsible for transporting the nutrients and oxygen a fetus needs to develop and grow. Alterations in placental formation and function, therefore, have consequences for fetal growth and birthweight, which in turn determine perinatal survival and risk of non‐communicable diseases for the offspring in later postnatal life. However, the placenta is not a static organ. As this review summarizes, research from multiple species has demonstrated that placental formation and function alter developmentally to the needs of the fetus for substrates for growth during normal gestation, as well as when there is greater competition for substrates in polytocous species and monotocous species with multiple gestations. The placenta also adapts in response to the gestational environment, integrating information about the ability of the mother to provide nutrients and oxygen with the needs of the fetus in that prevailing environment. In particular, placental structure (e.g. vascularity, surface area, blood flow, diffusion distance) and transport capacity (e.g. nutrient transporter levels and activity) respond to suboptimal gestational environments, namely malnutrition, obesity, hypoxia and maternal ageing. Mechanisms mediating developmentally and environmentally induced homeostatic responses of the placenta that help support normal fetal growth include imprinted genes, signalling pathways, subcellular constituents and fetal sexomes. Identification of these placental strategies may inform the development of therapies for complicated human pregnancies and advance understanding of the pathways underlying poor fetal outcomes and their consequences for health and disease risk.

## Abstract (highlights)

- **What is the topic of this review?**
  How the placenta, which transports nutrients and oxygen to the fetus, may alter its support of fetal growth developmentally and with adverse gestational conditions.
- **What advances does it highlight?**
  Placental formation and function alter with the needs of the fetus for substrates for growth during normal gestation and when there is enhanced competition for substrates in species with multiple gestations or adverse gestational environments, and this is mediated by imprinted genes, signalling pathways, mitochondria and fetal sexomes.

## 1 INTRODUCTION

The placenta is vital for mammalian development and a key determinant of life‐long health. It is the organ that forms the interface between the mother and the fetus and is responsible for transporting all the nutrients and oxygen a fetus needs to develop and grow. Expectedly, the inability of the placenta to grow and function properly may result in miscarriage, fetal growth restriction or stillbirth. There can also be a lasting impact on the risk to the child of developing life‐shortening conditions like cardio‐metabolic diseases, as a result of intrauterine developmental programming (Lewis &, Sferruzzi‐Perri, in press; Schroeder et al., 2022; Sferruzzi‐Perri & Camm, 2016). However, the placenta is not a static organ. Research conducted in humans and experimental animal models, including mice, rats, guinea pigs and sheep, has demonstrated that the placenta develops morphologically and functionally to meet the needs of the growing fetus for substrates during normal gestation. The placenta also adapts to the gestational environment and integrates information about the availability of maternal nutrients and oxygen with the needs of the fetus to regulate development in the prevailing conditions. The objective of this review is to summarize novel findings of the placental adaptations that support the increasing fetal growth demands during pregnancy in normal and suboptimal environmental conditions, including when there is sibling competition for substrates in polytocous and monotocous species. This review explores how placental structure and transport capacity respond to the gestational environments relevant to human pregnancy, explicitly malnutrition, obesity, hypoxia/high altitude and extremes of maternal age. Relevant findings for each gestational environment or condition will be discussed in relation to changes in placental morphological development and placental expression of genes for key nutrient transporters required for fetal growth, namely those which transport glucose (e.g., *Slc2a1*,*3*), fatty acids (e.g., *Fatp1*,*4*, *Fabp4*,*5* and *Cd36*), and amino acids (e.g., system L, including *Slc7a5*,*8* and system A, including *Slc38a1*,*2*,*4*, which transport large and small neutral amino acids, respectively). Finally, the review evaluates the contribution of imprinted genes, signalling pathways, subcellular constituents, and fetal sexomes and hormones as potential mechanisms mediating developmentally and environmentally induced responses of the placenta that help support fetal growth demands. The review focuses on data available from mice, rats, guinea pigs, rabbits and sheep, in addition to humans, where possible – where the majority of work has been performed. By adopting a comparative approach, the review will highlight common strategies driving placental adaptations in aforementioned mammalian species, and also provide potential targets for intervention in compromised human pregnancies.

## 2 ADAPTATIONS OF THE PLACENTA DURING NORMAL PREGNANCY

### 2.1 As fetal growth demands increase during normal pregnancy

In eutherian mammals, the placenta has achieved its greatest size, in terms of mass, by the last third of pregnancy, when the fetus enters its most rapid, exponential growth phase. To meet increasing fetal needs for oxygen and nutrients to grow, the placenta undergoes extensive morphological remodelling (Fowden et al., 2009). In particular, there is an arborization of placental villi, extended branching of the fetal capillaries within them, and thinning of the syncytiotrophoblast separating the maternal and fetal circulations in humans (Cindrova‐Davies & Sferruzzi‐Perri, 2022) – morphological features that correlate with birthweight (Salafia et al., 2008). Similar changes occur in the labyrinthine exchange region of the placenta in guinea pigs, rabbit, rats and mice – species that also have a haemochorial placenta like humans (Adamson et al., 2002; Coan et al., 2004, 2005; De Clercq et al., 2019; Furukawa et al., 2019; McArdle et al., 2009; Roberts et al., 2001). Even sheep, which have a synepitheliochorial placenta (due to the retention of the uterine epithelium lying between the maternal and fetal blood), display increased elaboration of maternal crypts, enhanced fetal villus branching and greater interdigitation between the two circulations as gestation advances (Borowicz et al., 2007; Hafez et al., 2010; Reynolds et al., 2005). These changes serve to increase the area available for substrate exchange, enhance delivery of substrates to and from the placenta, and reduce the diffusion distance for molecules, including oxygen, to traverse from mother to fetus in the placenta. There is also increased expression and activity of specific glucose, amino acid and fatty acid transporters in the placenta in normal, healthy human (James‐Allan et al., 2020; Magnusson‐Olsson et al., 2006; Mahendran et al., 1994), rabbit (Khan et al., 2011; Lopez‐Tello, Arias‐Alvarez, et al., 2019), rat (Jansson et al., 2006), and mouse pregnancy (Coan et al., 2008; Sferruzzi‐Perri et al., 2011). Finally in sheep, placental capacity for glucose transport increases in the second half of pregnancy (Bell & Ehrhardt, 2002).

### 2.2 Within‐litter variability related to placental or fetal size in polytocous species and multiple pregnancies

In humans, studies of twin pregnancies have shown that placental vascular density and surface area are greater for the twin with heavier birthweight (Freedman et al., 2019; Voicu et al., 2020). Moreover, compared to singletons, placental vascular density is increased in twin pregnancies (Voicu et al., 2020). In mice, morphological characterization of a fused placenta supporting fetuses with growth discordance also found improved vascularization, as well as a thinner trophoblast barrier for diffusion of the labyrinthine zone supporting the heavier of the two fetuses (Lopez‐Tello & Sferruzzi‐Perri, 2021). There are also more everted placentomes that have increased placental nutrient transport capacity in twin versus singleton sheep gestations (van der Linden et al., 2013). Overall, these data indicate that there may be adaptations occurring in the placental vascular bed to support fetal growth when the maternal ability to provide substrates may be constrained in multiple gestations.

Studies conducted in mice comparing the lightest versus the heaviest placenta in the litter show a relative enlargement of the labyrinthine zone and increased transport of amino acids (system A and X_AG‐_) and calcium in vivo (Coan et al., 2008; Hayward et al., 2018, 2017; McIntyre et al., 2019). Recent work instead comparing the placenta supporting the lightest to the heaviest fetuses of the litter in mice showed lower maternal blood space volume and surface area for females and lower glucose transport ability in males (Salazar‐Petres et al., 2022). In the guinea pig, maternal blood flow is also the lowest for placentas supporting the lightest fetuses in the litter, but findings also suggest that placentas of the heaviest fetuses may be hyperperfused (Myers et al., 1982). Taken together, these results suggest that morphological and functional adaptive responses of the placenta may be influenced by fetal sex and that placental nutrient transport capacity and blood perfusion are critical factors in the regulation of fetal growth.

### 2.3 Adaptations of the placenta during adverse gestational environments

The adaptive capabilities of the placenta are likely to be most important and evident when the gestational environment is unfavourable. The impact of unfavourable gestational environments, namely maternal malnutrition, obesity, reduced oxygen availability (hypoxia) and extremes of age on placental structural and functional changes in relation to fetal growth are shown in Tables 1, 2, 3, 4 and summarized below. Overall, the nature of the changes seems to depend on the type, timing, severity of the unfavourable environment, as well as the specific species studied.

**TABLE 1:** Adaptations in the placenta in response to malnutrition.

Maternal undernutrition using small animal models								
			Placenta					
Animal species	Intervention	Fetal outcome	Size, morphology, and blood flow	Transport function in vivo	Transporters	Imprinted genes	Subcellular components and signalling pathways	References
Mouse	50% caloric restriction prior to pregnancy							
	50% caloric restriction between 3 weeks prior to pregnancy through GD11.5	GD18.5: ↔ FW	GD18.5: ↔PW GD18.5: ↔LZ size, ↑MBS		GD18.5: ↓*Slc38a4*	GD18.5: ↓*Slc38a4*		Van Gronigen Case et al. (2021)
	50% caloric restriction during pregnancy							
	Restricted between days 10 and 18.5	GD18.5: ↓FW	GD18.5: ↓PW	GD18.5: ↓Glucose and system L transport ↑System A transport	GD18.5: ↓*Slc2a3*, *Slc7a8* ↔ *Slc2a1*, ↑*Slc38a1*, ↑*Slc38a2*		↓mTOR and ↔AMPK signalling	Ganguly et al. (2012)
	Restricted between days 10 and 19	GD18.5: ↓FW	GD18.5: ↓PW	GD18.5: ↓Glucose ↓Leucine ↓Blood flow	GD18.5: ↓*Slc2a3* ↔ *Slc2a1*, ↓*Slc7a8*		↑5‐hmC, KDM3A, and *miRNAs‐149*	Ganguly et al. (2016)
	Restricted between days 1.5 and 11.5	GD11.5: ND	GD11.5: ↓PW, ↑Lz/Jz and ↓fetal area in Lz				↓*Prl8a8, Tpbpa, Ceacam12* and *Gbp1* Integral to membrane ∆Collagen ∆Metal binding ∆Haem binding ∆Cell motility	Schulz et al. (2012)
	Restricted between days 10 and 19	GD19: ND	GD19: ND		GD19: ↓*Slc2a3* ↓*Slc7a8*	GD19: ↑*Ptger1*	↓Global methylation (↓DNMT1) ↑*miR‐149*	Chen et al. (2013)
	Restricted between days 1.5 and 11.5	GD12: ↔FW	GD12: ↔PW, Lz area					Harper et al. (2015)
	25% caloric restriction during pregnancy							
	Restricted between days 10 and 19	GD19: ↓FW	GD19: ↔PW	GD19: ↑Glucose and ↓system L transport	GD19: ↑*Slc2a3*		↑*miR‐10b* ↑5‐hmC, and KDM3A	Ganguly et al. (2016)
	20% caloric restriction during pregnancy							
	Restricted between days 3 and 19	GD16: ↔FW GD19: ↓FW	GD16: ↓PW GD19: ↓PW GD16: ↔Lz size and morphology GD19: ↓Lz size, ↓MBS, FC, SA	GD16: ↔MeGlu, MeAIB GD19: ↔MeGlu ↑MeAIB	GD16: ↓*Slc2a1* GD19: ↑*Slc2a1*, ↑*Slc38a2*	GD16: ↓*Igf2P0* GD19: *↓Igf2P0* ↓*Slc38a4*	GD16: ↑IGF1R, ↓PI3K signalling GD19: ↔IGF1R, ↓PI3K signalling	Coan et al. (2010), Sferruzzi‐Perri et al. (2011)
Rat	50% caloric restriction during pregnancy							
	Restricted between days 5 and 20/21	GD20‐21: ↓FW	GD20‐21: ↓PW	↓System A uptake				Ahokas et al. (1983, 1981)
	Restricted between days 10 and 20	GD20: ↓FW	GD20: ↓PW, ↓Lz weight		GD20: ↓*Slc2a3*, ↓*Slc38a1*, ↓*Slc38a2*	GD20: ↑*Slc38a4*	↑Apoptosis ↓BCL2 and BCL‐X_L_ ↑Cytochrome *c*, caspase 9 and 3 ↓PPARγ	Belkacemi, Desai, et al. (2011), Belkacemi, Jelks, et al. (2011)
	Restricted between days 10 and 20	GD21: ↓FW	GD21: ↔PW		GD21: ↓*Slc2a3*			Lesage et al. (2001, 2002)
Rabbit	50% food intake during pregnancy							
	Restricted between days 9 and 28	GD21: ↓FW GD28: ↓FW	GD21: ↓PW ↓Length GD28: ↓PW ↓Size, length, thickness ↑Fibrosis				↑Apoptosis	Lopez‐Tello et al. (2017, 2015)
Sheep	50% total energy intake, 50% food intake, 50% dietary requirements							
	Restricted between days 28 and 78	GD78: ↓FW	↔Number of placentomes ↑Maternal caruncle vascularity					Vonnahme et al. (2003)
	Restricted between days 28 and 77	GD145: ↔	GD145: ↑PW ↑Fetal cotyledon weight ↔Maternal caruncle weight ↓Type A placentomes ↑Type B placentomes					Heasman et al. (1998)
	Restricted between day 28 and 78	GD78: ↓FW GD135: ↔FW	GD78: ↓PW GD135: ↔PW		GD78: ↑FATP4, GLUT1, CD36		↑AMPK, ACC, ERK signalling ↔mTOR, AKT ↓Leptin	Ma et al. (2011)
	Restricted between days 35 and 125	GD125: ↔FW (all restricted lambs) or ↓FW (only IUGR lambs)	GD125: ↓PW ↓SA (only IUGR lambs)		GD125: ↓*Slc7a2*, *Slc7a6, Slc7a7, Slc38a2* ↔*Slc7a5, Slc7a8* (only IUGR lambs)			Edwards et al. (2020)
	Restricted between 60 days before mating and 30 days after	GD78: ↔FW	GD78: ↓PW ↑Maternal caruncle vascularity ↑Fetal cotyledon vascularity				↑ERK 1/2, AKT phosphorylation in cotyledon ↔ERK1/2, AKT phosphorylation in caruncle	Zhu et al. (2007)
	40% reduction							
	Restricted from 30 days before mating (UNPre), 50 days after mating (UnPost) or during both periods (UNB)	GD75: ↓FW (UNB and UNPost) ↔FW in UNPre	↓PW (UNB) ↑Type A placentome (UNB) ↓Type B placentome (UNB)					Macias‐Cruz et al. (2017)
	Restricted between day 28 and 80	GD80: ↔FW	GD80: ↓PW		GD80: ↔*Slc2a1*			Dandrea et al. (2001)
	Acute nutrient restriction							
	Reduced from day 83 and withdrawn between day 85 and 90	GD90: ↔FW GD135: ↔FW	GD90: ↓PW GD135: ↔PW ↑Type D placentome		GD90: ↓*Igfbp3*, *Vegf*, ↔*Slc2a1, Slc2a3* GD135: ↓*Igfbp2*			McMullen et al. (2005)
	15% reduction							
	Restricted between 14 days before mating to GD70	GD130: ↔FW	GD130: ↔PW Shift between A and D‐type placentomes					Steyn et al. (2001)
Guinea pig	15% caloric restriction							
	Restricted between 151 days before pregnancy to GD61	GD61: ↓FW	GD61: ↓PW ↓MBS, FC		GD61: ↓P‐			Soo et al. (2012)
	10−30% caloric restriction							
	Restricted between day 28 before pregnancy and throughout pregnancy	GD30, 40 and 60: ↓FW	GD30,40 and 60: ↓PW GD30: ↔Lz size ↓MBS, SA ↑BT GD60: ↓Lz size ↓TBS, MBS, FC, SA ↑BT			↓*Igf2*		Olausson and Sohlstrom (2003), Roberts et al. (2001), Sohlstrom, Katsman, Kind, Grant, et al. (1998), Sohlstrom, Katsman, Kind, Roberts, et al. (1998)

↑: increase; ↓: decrease; ↔: no change; ∆ altered. Abbreviations: 5‐hmC, 5‐hydroxymethylcytosine; ACC, Acetyl‐CoA carboylase; AMPK, 5' AMP‐activated protein kinase; BCL2, B‐cell lynphoma 2; BT, barrier thickness; *Ceacam12*, carcionoembryonic antigen 12; DNMT1, DNA(cytosine‐5)‐methyltransferasa; ERK1/2, extracelluar signal‐regulated kinase 1/2; FATP4, fatty acid transporter 4; FC, fetal capillaries; FW, fetal weight; *Gbp1*, guanylate binding protein 1; GD, gestational day; IGF1R, insulin growth factor 1 receptor; *Igf2*, insulin growth factor; *Igfbp2*, insulin like growth factor binding protein 2; *Igfbp3*, insulin like growth factor binding protein 3; *Igf2P0*, placental specific insulin growth factor 2;  IUGR, intrauterine growth restriction; Jz, junctional zone; KDM3A, lysine demethylase 3A; Lz, labyrinthine zone; MeAIB, methyl amino isobutyric acid (system A transport); MeGlu, methyl d‐glucose; MBS, maternal blood spaces; mTOR, mammalian target of rapamycin; ND, not determined; PPARγ, peroxisome proliferator‐activated receptor gamma, PI3K, phosphoinositide 3‐kinase; *Prl8a8*, prolactin family 8, subfamily a, member 8; *Ptger1*, prostaglandin E receptor 1; PW, placental weight; SA, surface area; *Slc2a1*, solute carrier family 2 member 1; *Slc2a3*, solute carrier family 2 member 3; Slc7a2, solute carrier family 7 member 2; Slc7a5, solute carrier family 7 member 5; Slc7a6, solute carrier family 7 member 6; Slc7a7, solute carrier family 7 member 7; *Slc7a8*, solute carrier family 7 member 8; *Slc38a1*, solute carrier family 38 member 1;*Slc38a2*, solute carrier family 38 member 2; *Slc38a4*, solute carrier family 38 member 4; TB, trophoblast; *Tpbpa*, trophoblast‐specific protein alpha.

**TABLE 2:** Adaptations in the placenta in response to obesity.

Maternal overnutrition using small animal models								
			Placental					
Animal species	Intervention	Fetal outcome	Size, morphology, and blood flow	Transport function in vivo	Transporters	Imprinted genes	Subcellular components and signalling pathways	References
Mouse	HFD (2.31× fat) 12 weeks prior to mating and throughout gestation	GD18.5: ↓FW (female and males)	GD18.5: ↔PW GD12.5 and 18.5: ↓vessel density (males)				↑ET‐1 signalling pathway ↑p38MAPK signalling ↑Leptin signalling	Stuart et al. (2018)
	HFD (2.5× fat) 8 weeks prior to mating and throughout gestation	GD15.5: ↔FW GD17.5: ↑FW	GD15.5: ↑PW ↓Proliferation GD17.5: ↔PW ↓Lz and proliferation				↑Cytokine expression (*IL6*, *IL1b*, *Tnfα*, *IL10*) and macrophage activation	Kim et al. (2014)
	HFD (3× fat) 8 weeks prior to mating and throughout gestation	GD19: ↑FW	GD19: ↔PW	GD19: ↑MeGlu ↑MeAIB	GD19: ↑SLC2A1, SNAT2 ↔SLC2A3, SNAT4			Jones et al. (2008)
	HFD (3.45× fat) 6 weeks prior to mating and throughout gestation	GD19: ↓FW	GD19: ↓PW ↓Lz ↓TB, MBS, SA ↓FC length, diffusion capacity ↑FC diameter, BT				↑DRP1, ACC activation (males) ↑PGC1α, PPARγ (female) ↓phospho‐AMPK (males) ↑Mito CII, ATPase (males) ↓UCP2 (male and female)	Napso et al. (2022)
	HSHF (5.14× sugar and 3.45× fat) during gestation until days 16 and 19	GD16: ↓FW GD19: ↔FW	GD16: ↓PW GD19: ↓PW	GD16: ↑ MeGlu, ↑ MeAIB	GD16: ↑*Slc2a3*, *Slc8a2* GD19: ↑FATP	GD16: ↑*Igf2*, *H19*, *Dlk1*, *Snrpn*, *Phlda2*, *Grb10*	GD16: ↑PI3K‐p110α, phospho‐AKT (S473), p70S6K total, MAPK total, phospho‐GSK3 (S9) GD19: ↑PI3K‐p110α, phospho‐AKT (S473), phospho‐GSK3 (S9), phospho‐MAPK, ↓4EBP1 total	Sferruzzi‐Perri et al. (2013)
	HSHF (4.7× sugar and 3.45× fat) 3 weeks prior to mating and throughout gestation	GD18.5: ↔FW	GD18.5: ↔PW ↑Glycogen cells number (females)					Lean et al. (2022)
	HFD (3.7× fat) during pregnancy	GD16: ↑FW	GD16: ↑PW ↑Blood sinusoids area		GD16: ↑*Fatp1* ↑FATP4, SNAT2		↑Proliferation	Song et al. (2018)
	HFD (3.7× fat) 12 weeks prior mating	GD16: ↓FW	GD16: ↔PW ↓Blood sinusoids area		GD16: ↓*Fatp1*, *Slc38a2*		↓Proliferation	Song et al. (2018)
	HFD (3.7× fat) 12 weeks prior mating and during pregnancy	GD16: ↔FW	GD16: ↔PW ↔Blood sinusoids area					Song et al. (2018)
	HFD (3.75× fat) during pregnancy	GD18: ↑FW	GD18: ↑PW		GD18: ↓SLC2A3		↑NOV/CCN3 signalling mTOR	Wang et al. (2021)
	HFD (4.61× fat) 4 weeks prior pregnancy	GD18.5: ↔FW	GD18.5: ↑PW Lz architectural distortion ↑Lipid deposition				↓*Cpt2* ↔*Cpt1a*, *Lcad*, *Lchad*, *Ampk* ↓*Sirt1*, *Pgc1α*, *Pparg*, *Tfam* ↓phospho‐AMPKα	Zhang et al. (2022)
	HFD (3.5× fat) 6 weeks prior pregnancy	GD14.5: ↔FW	GD14.5: ↔ PW ↑VEGF, CD31 (male and female) ↓α‐SMA		↑*Slc2a1*, *Slc2a3*, *Slc38a2* ↔*Fabp4*		↑*Nfkb* (females), ↑*Tnf*, *IL6*, *Mcp1* (males) ↑carbonic anhydrase IX (marker of hypoxia) (male and female)	Wallace et al. (2019)
	HFD (6× fat) during pregnancy	GD15: ↔FW (male and female)	GD15: ↑PW (male and female) ↔Lz vascularity		↓*Slc22a1*, *Dlk1*, *Dio3, Slc22a1* (females) ↑*Slc22a2* (males)		Sex‐specific epigenetic alterations within CpG and several dysregulated biological functions by IPA analysis	Gabory et al. (2012), Gallou‐Kabani et al. (2010)
	HFD (6.6× fat) Between females weaning until pregnancy days 15.5 and 18.5	GD15.5: ↓FW GD18.5: ↔FW	GD15.5: ↔PW ↓Lz/Placenta volume ↓Trophoblast differentiation (E‐cadherin)	GD15.5: ↑Mannitol	GD15.5: ↑SLC2A4 GD18.5: ↑SLC2A1, SLC2A4		↑Lipid accumulation ↑Lipoprotein deposition ↓Wnt–GSK3β signalling	Appel et al. (2019), Kretschmer et al. (2020)
	HFD (12× fat) 4 weeks prior mating and during pregnancy	GD19: ↔FW	GD19: ↓PW ↓Trophoblast number, ↑Dead endothelial cells				↑Endothelial apoptosis ↑Lipid peroxidation, oxidative stress	Liang et al. (2010, 2009)
	HFD (4.6× fat) 4 weeks prior mating and during pregnancy	GD12.5: ND	GD12.5: ND				Pathway analysis: cytoskeletal regulation (males) and Insulin/IGF/MAPK pathway (females)	Barke et al. (2019)
Rat	HFD (2.8× fat) 16 weeks prior mating and during pregnancy	GD15: ↓FW	GD15: ↔PW ↑Lz vascularization				↓Remodelling of maternal spiral arteries ↓Smooth muscle actin ↑Oxidative stress ↑Invasion (MMP9) ↑Inflammation (MCP‐1)	Hayes et al. (2012, 2014)
	HFD (4.4× fat) Between pregnancy days 2 and 21	GD21: ↔FW (males) ↓FW (females)	GD21: ↔PW ↓Thickness		GD21: ↑*Scll2a3*, *Slc38a2*, SNAT2 (males) ↔*Slc2a1*, *Slc38a1,4*		↑Total 4EBP1 (male) ↓AKT activation (male) ↑IGF2R‐IGF2 axis (female)	Song et al. (2017)
	HFD (5–6× fat) 7 weeks prior mating and during pregnancy	GD21: ↑FW	GD21: ↔PW		GD21: ↓SNAT1 ↔SNAT2,4 ↔ SLC2A1,3,9 ↔FATP4		↑mTOR signalling ↓Phospho‐eIF2α ↓Phospho‐AMPK ↔LPL abundance and activity ↔JNK, STAT3	Gaccioli et al. (2013)
	60% kcal fat on diet during pregnancy	GD18.5: ND	GD18.5: ND ↓Lz sinusoid areas ↓Placental efficiency				↓SIRT1, PGC1α, VEGF (both protein and gene expression)	Zhu, Du, et al. (2010)
Rabbit	HFD (4× fat) 8 weeks prior mating and during pregnancy	GD28: ↓FW (female and male)	GD28: ND ↑Lipid droplets ↔Total cholesterol ↑Cholesteryl esters		GD28: ↔*Slc2a1*,*3* ↔*Slc38a2*,*4* ↓*Slc38a1*, *Ldl‐r*, *Cd36*, *Lxr‐a*			Tarrade et al. (2013)
Sheep	High palatable diet (1.5×, 60 days prior mating until 75 or 135 days of gestation	GD75: ↑FW GD135: ↔FW	GD75: ↔Total placentome number ↔Total placentome weight ↔Average placentome weight ↓Cotyledonary vessel area density ↑Cotyledonary arteriole diameter GD135: ↔Total placentome number ↓Total placentome weight ↓Average placentome weight		GD75: ↑FATP1 (protein and mRNA), FATP4 (protein), *Cd36* (mRNA) ↔ *Fabp1,3–5* GD135: ↑*Fatp1*, FATP4 (protein and mRNA), *Cd36*		GD75: ↑PPARγ (protein and mRNA), ↔p38MAPK activation in COT tissue ↓VEGF, FGF2, PLGF, ANG1, ANG2 (protein and mRNA) HIF‐1 (mRNA) in COT arterial tissue ↓PLGF, ANG1, and ANG2 (protein) in COT tissue GD135: ↑ANG‐2 in COT arterial tissue ↓Total mTOR, ERK1/2, AMPK, ACC ↓Phosphorylated AKT, mTOR, ERK1/2, ACC, IR	Ma et al. (2010), Zhu et al. (2009), Zhu, Ma, et al. (2010)

↑: increase; ↓: decrease; ↔: no change. Abbreviations: 4EBP1, eukaryotic translation initiation factor 4E‐binding protein 1, ACC, Acetyl‐CoA carboylase; AKT, protein kinase B, AMPK, 5' AMP‐activated protein kinase; ANG1, angiopoietin 1; ANG2, angiopoietin 2; BT, barrierthickness; CII, complex II; *Cpt1a*, carnitine palmitoyltransferase 1alpha; *Cpt2*, carnitine palmitoyltransferase 2; *Dio3*, iodothyronine deiodinase 3; *Dlk1*, delta like non‐canonical notch ligand 1; DRP1, dynamyn‐related protein 1; ET‐1, endothelin‐1; ERK1/2, extracelluar signal‐regulated kinase 1/2; *Fabp1*, fatty acid binding protein 1; *Fabp3*, fatty acid binding protein 3; *Fabp5*, fatty acid binding protein 5; FATP, fatty acid transporter; *Fatp1*, fatty acid transporter 1; FATP4, fatty acid transporter 4; FC, fetal capillaries; FGF2, fibroblast growth factor 2; FW, fetal weight; GD, gestational day; GSK3, glycogen synthase kinase 3; *Grb10*, growth  HFD, high‐fat diet; HIF‐1, hypoxia‐inducible factor 1; *Igf2*, insulin growth factor 2; IL, interleukin; IR, insulin receptor; JNK, c‐Jun N‐terminal kinase; *Lcad*, long‐chain acyl‐CoA dehydrogenase; *Lchad*, long‐chain 3‐hydroxyacyl‐CoA dehydrogenase; *Ldlr*, low density lipoprotein receptor; *Lxra*, liver X receptor alpha; Lz, labyrinthine zone; *Mcp1*, monocyte chemoattractant protein‐1; MeAIB, methyl amino isobutyric acid; MeGlu, methyl d‐glucose; MBS, maternal blood spaces; mTOR, mammalian target of rapamycin; *Nfkb*, nuclear factor kappa‐light‐chain; ND, not determined; NOV/CCN3, nephroblastoma overexpressed; p38‐MAPK, p38 mitogen‐activated protein kinase; PGC1α, peroxisome proliferator‐activated receptor gamma coactivator 1‐alpha; *Phlda2*, pleckstrin homology like domain family A member 2; PLGF, placental growth factor; PPARγ, peroxisome proliferator‐activated receptor gamma; PW, placental weight; p70S6K, ribosomal protein S6 kinase beta‐1, SA, surface area; *Sirt1*, sirtuin 1; SLC2A1; solute carrier family 2 member 1; SLC2A3; solute carrier family 2 member 3; SLC2A4; solute carrier family 2 member 4; *Slc38a1*, solute carrier family 38 member 1; *Slc38a2*, solute carrier family 38 member 2; *Slc38a4*, solute carrier family 38 member 4;  SNAT2, sodium‐coupled neutral amino acid transporter 2; SNAT4, sodium‐coupled neutral amino acid transporter 4; *Snprn*, small nuclear ribonucleoprotein polypeptide N; STAT3, signal transducer and activator of transcription 3; TB, trophoblast; *Tfam*, mitochondrial transcription factor A; UCP2, mitochondrial uncoupling protein 2; VEGF, vascular endothelial growth factor.

**TABLE 3:** Adaptations in the placenta in response to hypoxia.

Maternal hypoxia using small animal models								
			Placental					
Animal species	Intervention	Fetal outcome	Size, morphology, and blood flow	Transport function in vivo	Transporters	Imprinted genes	Subcellular components and signalling pathways	References
Mouse	13% hypoxia between pregnancy day 1 and 19	GD18.5: ↓FW (female and male)	GD18.5: ↑PW (male) ↔Lz ↑Blood spaces ↑Maternal venous blood spaces ↑Maternal arterial and venous blood space				↑AKT (S473‐P), 4EBP‐1 and elF2a activation ↔AMPK activation ↓elF2a total protein levels ↑XBP‐1 HSP27, HSP60 ↔GRP78 ↑Oxidative stress ↓Complex III, V	Matheson et al. (2015)
	13% hypoxia between pregnancy days 11 and 16 and 14 and 19	GD16: ↔FW GD19: ↓FW	GD16: ↔PW ↑Lz, MBS, TB, SA GD19: ↔PW ↑FC ↓BT	GD16: ↔MeGlu ↔MeAIB GD19: ↑MeGlu ↔MeAIB	GD16: ↔*Slc2a1, Slc2a3* ↔*Slc38a1* GD19: ↑*Slc38a1*	GD19: ↑*Igf2, Igf20*	GD16: ↑AKT‐T308‐P, ↓AKT‐S473‐P. ↑PGC1α, ↑Oxidative stress ↔UCP2, ↓Complex III, ATP synthase, ↔protein carbonylation GD19: AKT‐S473‐P ↓IR, IGF1R, PI3K‐p85, PI3K‐p110α, AKT total. ↑Citrate synthase, PGC1α, ATP synthase, Complex IV, ↓fatty acid oxidation (under Leak and Oxphos)	Higgins et al. (2015), Sferruzzi‐Perri et al. (2019)
	12% hypoxia between pregnancy days 14 and 19	GD19: ↓FW (female and male)	GD19: ↔PW ↓Lz blood spaces		GD19: ↓*Slc2a1* (female) ↑*Slc38a1* (female) ↔*Slc2a3*	GD19: ↓*Igf2* (female)	↓*Hsd11b2* (female), ↓*Igf1r* (female), ↓*Nr3c2* (female)	Cuffe et al. (2014)
	11% hypoxia between pregnancy days 14.5–17.5	GD17.5: ↔FW	GD17.5: ↔PW ↓Lz vessel segments and vascular volume ↑Capillary tuft length and diameter ↓BT				↓Total utero‐placenta arterial resistance	Cahill et al. (2018)
	10% hypoxia between pregnancy days 14−19	GD19: ↓FW	GD19: ↔PW ↓Lz, MBS, SA ↑TB, BT	GD19: ↔MeGlu ↓MeAIB			↓AKT‐S473‐P, PI3K‐p110β ↓Fatty acid oxidation (under Leak and Oxphos) ↑Citrate synthase ↑Protein carbonylation ↑AMPK signalling	Higgins et al. (2015), Sferruzzi‐Perri et al. (2019), Skeffington et al. (2015)
Rat	13% hypoxia between pregnancy days 6 and 20	GD20: ↔FW	GD20: ↑PW ↑Lz volume ↑FC SA				↑HSP70, HNE ↓HSP90 ↔eNOS, Mn‐SOD ↑Catalase ↑GRP78, ATF4, GRP75, TID1	Nuzzo et al. (2018), Richter et al. (2012)
	9% hypoxia between pregnancy days 14.5 and 17.5	GD18.5: ↓FW	GD18.5: ↓PW				↑*mTOR, Irs1, Pik3r1, Ppp2r2b, Prkag3, Rps6ka2, Vegfc* ↓*Prkag2* ↓mTOR protein ↑Caspase 3	Kimball et al. (2015)
Guinea pig	12% hypoxia during gestation	GD64: ↔FW	GD64: ↔PW ↑Diffusion capacity ↑Vascular volume					Bacon et al. (1984)
	10.5% hypoxia between pregnancy days 50 and 64	GD64: ↓FW (female and male)					↑IGF2, phospho and total AKT, and PCNA (females)	Elsamadicy and Thompson (2022)
Maternal hypoxia using large animal models								
Sheep	10% hypoxia between pregnancy days 105−138	GD138: ↓FW	GD138: ↔PW ↑Fetal brain weight/fetal body weight ratio				↑JNK, ERK activation ↑HIF1α ↑Protein carbonylation ↑ATF6, UPR (ER + Cyt) ↑sFlt‐1, sEng, sFlt‐1/VEGF	Tong et al. (2022)
	High altitude (∼110 days)	GD140: ↔FW	GD140: ↔PW ↓Placentome number ↓Type A and ↑type B, C and D placentomes ↑Placental/brain weight ratio					Penninga and Longo (1998)

↑: increase; ↓: decrease; ↔: no change. Abbreviations: % Hypoxia, % of O_2_; 4EBP1, eukaryotic translation initiation factor 4E‐binding protein 1; AKT, protein kinase B; AMPK, 5' AMP‐activated protein kinase; ATF4, activating factor 4; ATF6, activating factor 6; BT, barrier thickness; Cyt, cytoplasmic; eIF2a, eukaryotic translation factor 2A; eNOS, endothelial nitric oxide synthase, ER, endoplasmic reticulum; ERK, extracelluar signal‐regulated kinase; FC, fetal capillaries; FW, fetal weight; GD, gestational day; GPR78, G Protein‐coupled receptor 78; GPR75, G Protein‐coupled receptor 75; HIF1α, hypoxia inducibe factor 1 alpha; *Hsd11b1*, hydroxysteroid 11 beta dehydrogenase 1; *Hsd11b2*, HSP27, heat shock protein 27; HSP60, heat shock protein 60; HSP70, heat shock protein 70; HSP90, heat shock protein 90; IGF1R, insulin growth factor 1 receptor; *Igf2*, insulin growth factor 2; *Igf2P0*, placental specific insulin growth factor 2; *Irs1*, insulin receptor substrate 1; JNK, c‐Jun N‐terminal kinase; Lz, labyrinthine zone; MBS, maternal blood spaces; MeAIB, methyl amino isobutyric acid; MeGlu, methyl d‐glucose; Mn‐SOD, manganese superoxide dismutase; mTOR, mammalian target of rapamycin; Nr3c2, nuclear receptor subfamily 3 group C member 2; PCNA, proliferating cell nuclear antigen; PGC1α, peroxisome proliferator‐activated receptor gamma coactivator 1‐alpha; PI3K‐p85, phosphoinositide 3‐kinase, p85 regulatory subunit; PI3K‐p110α, phosphoinositide 3‐kinase,  regulatory subunit; *Pik3r1*, phosphatidylinositol 3‐kinase regulatory subunit alpha;  PW, placental weight; SA, surface area; sENG, soluble endoglin; *sFlt‐1*, soluble fms‐like tyrosine kinase‐1; *Slc2a1*, solute carrier family 2 member 1; *Slc2a3*, solute carrier family 2 member 3; *Slc38a1*, solute carrier family 38 member 1; TB, trophoblast; TID1, tumurous imaginal disc 1; UCP2, mitochondrial uncoupling protein 2; UPR, unfolded protein response; VEGF, vascular endothelial growth factor; *Vegfc*, vascular endothelial growth factor C; XBP‐1, X‐box binding protein 1.

**TABLE 4:** Adaptations in the placenta in response to advanced age.

Advanced maternal age using small animal models							
			Placental				
Animal species	Intervention	Fetal outcome	Size, morphology, and blood flow	Transport function in vivo	Transporters	Subcellular components and signalling pathways	References
Mouse	38–41 weeks dams were studied at pregnancy day 17.5	GD 17.5: ↓FW ↓Viability	GD 17.5: ↑PW ↓Fetal/placental weight ratio	GD 17.5: ↓MeAIB and Taurine			Lean et al. (2017)
Rat	Aged versus young dams (2.5× older)	GD20: ↓FW (male and female)	GD20: ↔PW ↑Lz (female) ↓Fetal/placental weight ratio (female and male) ↑Glycogen cells (male) ↑Spongiotrophoblast		↔*Slc2a1,3* *Slc38a1*,*2*,*4*	↓*Hsd11b1* ↑*Hsd11b2* (females) ↔*Hsd11b1* ↓*Hsd11b2* (males) ↑*Igf2* (females), ↑*Prl3b1* (females) ↓*Vegf* (males) ↓IGF2 (males), ↓VEGF (both) ↑Oxidative stress (both) ↓Catalase (female) ↑Caspase 3 (male)	Napso et al. (2019)

↑: increase; ↓: decrease; ↔: no change. Abbreviations: FW, fetal weight; GD, gestational day; *Hsd11b1*, hydroxysteroid 11‐beta dehydrogenase1; *Hsd11b2*, hydroxysteroid 11‐beta dehydrogenase 2; *Igf2*, insulin growth factor 2; MeAIB, methyl amino isobutyric acid; Prl3b1, prolactin family 3, subunit b, member 1; PW, placental weight; *Slc2a1*, solute carrier family 2 member 1; *Slc2a3*, solute carrier family 2 member 3; *Slc38a1*, solute carrier family 38 member 1; *Slc38a2*, solute carrier family 38 member 2; *Slc38a4*, solute carrier family 38 member 4; VEGF, vascular endothelial growth factor.

### 2.4 Malnutrition

#### 2.4.1 Changes in placental structure

Malnutrition imposed by the Dutch Hunger Famine was associated with changes in placenta size and fetal weight, but the timing of the exposure determined the nature of effect (Lumey, 1998; Roseboom et al., 2011). For example, women exposed to famine during the pre‐conception period and first trimester of pregnancy exhibited compensatory increased placental growth and delivered normal birthweight babies when compared to pre‐famine, normally nourished control women (Lumey, 1998). Animal models have revealed that there are different morphological changes in the placenta that also link to fetal growth outcomes with maternal malnutrition (Table 1). In sheep, a 50% reduction of the total energy intake also leads to beneficial placental changes, namely increased caruncle vascularity in mid‐gestation, enhanced cotyledon growth, maturation and placental weight in late gestation, and normal fetal weight at term (Heasman et al., 1998; Vonnahme et al., 2003; Zhu et al., 2007). In sheep, a graded reduction in food intake across pregnancy (McMullen et al., 2005) or 15% reduction in food intake just in the first half of pregnancy (Steyn et al., 2001) also results in the formation of more mature placentas and preserved fetal weight. In mice, a 50% reduction in maternal food intake from prior to pregnancy is associated with no change in placental weight, preserved labyrinthine zone size, expanded placental–maternal blood spaces and fetal growth maintenance until term (Chen et al., 2013; Ganguly et al., 2012, 2016; Harper et al., 2015; Schulz et al., 2012; Van Gronigen Case et al., 2021). Also in mice, a 20% reduction in maternal food intake from the start of pregnancy is also accompanied by maintenance of labyrinthine zone size and fetal weight, at least until late gestation (Coan et al., 2010; Sferruzzi‐Perri et al., 2011).

However, not all changes in placental structure may operate to optimize or maintain fetal growth in the context of maternal undernutrition (Table 1). In rats and rabbits, a 50% reduction in maternal food intake during pregnancy leads to decreased placental labyrinthine zone size and increased placental cell apoptosis in association with fetal growth restriction (Ahokas et al., 1983, 1981; Belkacemi, Desai, et al., 2011; Belkacemi, Jelks, et al., 2011; Lesage et al., 2001, 2002; Lopez‐Tello et al., 2017, 2015). In guinea pigs, 10−30% restriction in maternal food intake from 4 weeks prior to pregnancy and then during pregnancy results in morphological defects in placental labyrinthine zone formation, including decreased fetal capillarization, increased barrier thickness and fetal growth restriction (Roberts et al., 2001; Sohlstrom, Katsman, Kind, Grant, et al., 1998; Sohlstrom, Katsman, Kind, Roberts, et al., 1998; Soo et al., 2012). In sheep, a 50% reduction in ewe dietary requirements from early through to late gestation causes reductions in placental volume and exchange surface area that are coupled with fetal growth restriction (Edwards et al., 2020). Finally, in twin‐bearing ewes, a 40% reduction in food intake leads to reductions in placental size, placental maturation and fetal growth (Macias‐Cruz et al., 2017). Thus, depending on the species, the timing and severity of the nutrient restriction, structural changes in placenta either serve to maintain and optimize fetal growth, or instead would be expected to limit substrate supply mediating the ensuing fetal growth restriction. However, it is important to mention that both types of placental adaptations would be beneficial in optimizing fetal survival and preserving maternal health by controlling the delivery of nutrients in the prevailing environment.

#### 2.4.2 Changes in placental function

System A activity is lower in the placenta of underweight versus normal‐weight women, who tend to have increased rates of babies that are small for gestational age (Hayward et al., 2012). There are also changes in glucose, amino acid and lipid transporter levels in the placenta that relate to maternal dietary or nutritional intakes (Brett et al., 2015, 2016). In animal models subjected to undernutrition, there are also changes in placental function, as informed by nutrient transport assays in vivo and nutrient transporter gene and protein levels, but changes are not always consistent (Table 1). In mice, a 20% caloric restriction from the beginning of pregnancy leads to adaptive upregulation of placental system A transport and increased *Slc38a2* and *Slc38a4* expression, with no change in glucose transport and maintained fetal growth until late gestation (Coan et al., 2010; Sferruzzi‐Perri et al., 2011). In mice exposed to a 50% reduction in maternal food intake from mid‐pregnancy, there is also adaptive upregulation of placental system A transport, elevated *Slc38a1* and *Slc38a2* expression and preserved fetal growth, even though glucose and system L amino acid transport, and *Slc2a3* and *Slc7a8* expression are compromised (Ganguly et al., 2012, 2016). Interpretations based on gene expression, namely *Fabp4* and *Fabp5* expression levels, indicate that maternal nutrient restriction may also result in adaptive upregulated placental lipid transport to the fetus. In rats, capacity for glucose transport, as indicated by decreased *Slc2a3* expression levels, is also reduced with 50% maternal undernutrition from mid‐pregnancy, but instead, system A transport and *Slc38a1* and *Slc38a2* expression are diminished and fetuses are growth‐stunted (Ahokas et al., 1981; Belkacemi, Jelks, et al., 2011; Lesage et al., 2002). However, in mice a 25% reduction in maternal caloric intake from mid‐gestation can result in increased *Slc2a3* expression and glucose transport, but this is associated with a secondary reduction in placental system L transport and fetal growth restriction (Ganguly et al., 2012, 2016). In undernourished mice, there is also a report of placental *Slc38a4* expression being less, but fetal weight unchanged compared to normally nourished controls (Van Gronigen Case et al., 2021). Last, findings relying on the expression or abundance of nutrient transporters in sheep have suggested that placental glucose and lipid transport capacity may be increased in early pregnancy by maternal undernutrition, as informed by *Slc2a1* and *Fatp4* expression levels, respectively, and these may relate to normal fetal growth in the model near term (Ma et al., 2011). In addition, unaffected fetal growth despite a 50% reduction in ewe food intake is related to the maintained expression of amino acid transporters, *Slc7a6*, *Slc7a8* and *Slc38a4*, by the placenta (Zhu et al., 2007). These data suggest that there may be a threshold for beneficial adaptive changes in specific nutrient transporter systems, and that different nutrient transporter systems can regulate and/or compensate for one another to thereby best support fetal growth.

### 2.5 Obesity

#### 2.5.1 Changes in placental structure

In women, maternal obesity can increase, decrease or not affect birthweight. Regardless, morphological changes like villus malformation, vascular defects and an increase in the thickness of the exchange barrier are reported (Bar et al., 2017; Rosado‐Yepez et al., 2020). In animal models, maternal obesity has been induced by feeding experimental diets containing high levels of sugar and/or fat (Table 2). In mice, diet‐induced obesity can compromise labyrinthine zone development, with reductions in the relative volume of the labyrinthine, decreased vessel formation, surface area and blood sinusoids, and increased barrier thickness in association with reduced fetal weight (Kretschmer et al., 2020; Napso et al., 2022; Peng et al., 2022; Song et al., 2018; Stuart et al., 2018). However, the specific nature of the effect can depend on fetal sex, with morphological defects often more pronounced in female versus male placentas (Napso et al., 2022; Stuart et al., 2018). There are also reports of decreased trophoblast differentiation, increased placental endothelial cell apoptosis and defective spiral artery remodelling in mice or rats also showing fetal growth restriction in response to maternal obesogenic diets (Baltayeva et al., 2020; Hayes et al., 2012, 2014; Kretschmer et al., 2020; Liang et al., 2010, 2009). In pregnant ewes, over‐feeding can also result in reduced cotyledon vascular density and a slowing of fetal growth towards term (Ma et al., 2010; Zhu et al., 2009). Placental thickness is also reduced in rats fed high‐fat diets (Song et al., 2017) and there are reports of disorganized labyrinthine zone structure in high‐fat‐fed mice (Zhang et al., 2022), even if fetal development may not always be impaired. Alterations in placental weight and labyrinthine zone morphology with an obesogenic diet can be linked to reduced neonatal viability (Lean et al., 2022). However, other studies showing changes in placental labyrinthine zone morphology have reported elevated fetal weight or increased fetal growth rate in late gestation in rodents fed obesogenic diets, again with the greatest changes seen for the male fetuses (Kim et al., 2014; Sferruzzi‐Perri et al., 2013). These data together suggest that changes in placental structure may contribute to changes in fetal growth trajectory with maternal obesity; however, the nature of the change may depend on the specific obesogenic diet regime used and the level of maternal obesity induced.

#### 2.5.2 Changes in placental function

There are also data from humans and experimental models showing that placental function is altered in response to maternal obesity, but the nature of the change relates to fetal growth outcomes (Table 2). In humans, placental capacity for amino acid and lipid transport is greater in obese compared to lean women who delivered babies of increased birth weight (Castillo‐Castrejon et al., 2019; Jansson et al., 2012). In contrast, woman delivering normal birth weight babies, placental lipid and amino acid transporter activity is reduced (Ditchfield et al., 2015; Farley et al., 2010; Segura et al., 2017). Similarly, in mice and rats fed obesogenic diets showing increased or accelerated fetal growth in late gestation, placental supply of glucose and system A, and placental glucose (*Slc2a1*, *Slc2a3*), amino acid (*Slc38a2*) and lipid (*Fatp1*, *Fatp4*) transporter gene or protein levels are increased (Jones et al., 2008; Sferruzzi‐Perri et al., 2013; Song et al., 2017). The nature of the effect can also depend on fetal sex in these obese rodent models (Song et al., 2017) and is influenced by the timing of the maternal obesogenic diet exposure (Song et al., 2018). Passive permeability of the placenta (informed by mannitol) and glucose transporters are also upregulated in line with enhanced late gestational fetal growth in response to maternal obesity (Appel et al., 2019; Kretschmer et al., 2020). In sheep that are obese through excess food intake, placental fatty acid transporters are upregulated in mid‐gestation when fetal growth is greater than controls (Zhu, Ma, et al., 2010). Moreover, in rabbits fed high‐fat diets, placental lipid and amino acid supply capacity is reduced in line with restricted fetal weight (Tarrade et al., 2013). However, diet‐induced maternal obesity can result in changes in placental nutrient transporter levels that do not seem to directly relate to fetal weight (Gabory et al., 2012). For instance, placental glucose transporter is reduced, but fetal growth increased in some models (Gaccioli et al., 2013; Wang et al., 2021), and glucose and amino acid transporters increased without a change, or even a decrease in fetal weight in others (Song et al., 2017; Wallace et al., 2019). Further work is required to assess the contribution of metabolic changes, like maternal glucose intolerance and dyslipidaemia, to the resultant effects of obesity on placental phenotype (Fernandez‐Twinn et al., 2017; Musial et al., 2019, 2017).

### 2.6 Hypoxia/high altitude

#### 2.6.1 Changes in placental structure

Deficiency in maternal oxygen availability can also affect placental phenotype and impact fetal outcomes. At high altitude, oxygen availability in the mother is reduced, and structural assessments of the delivered human placenta have found reduced volume and surface area of villi, but a thinner barrier to diffusion and greater villous capillarization that may optimize fetal growth (Ali et al., 1996; Espinoza et al., 2001; Jackson et al., 1988a, 1988b; Mayhew, 2003). In pre‐eclampsia where the placenta is mal‐perfused and hypoxic, villous maturation and surface area are decreased leading to reduced birthweight (Egbor et al., 2006; Mayhew et al., 2003; Teasdale, 1987). In rodents exposed to 13% oxygen (13% hypoxia), there are beneficial changes in placental labyrinthine zone morphology, namely increased vascular density or surface area, enlarged maternal blood spaces, and a thinner barrier to diffusion in association with a modest decrease or no change in fetal development (Higgins et al., 2015; Matheson et al., 2015; Nuzzo et al., 2018; Richter et al., 2012) (Table 3). The precise change, however, depends on the timing of the exposure (Higgins et al., 2015). For instance, 13% hypoxia between days 11 and 16 of pregnancy increased maternal blood volume and exchange area, whereas 13% hypoxia between days 14 and 19 increased fetal capillary volume and reduced the barrier to diffusion in the mouse placenta (Higgins et al., 2015). In guinea pigs, exposure to 12% oxygen (12% hypoxia) is also linked to increased placental vascular volume and reduced trophoblast diffusion distance and no change in fetal weight at term (Bacon et al., 1984). Furthermore, there are a greater number of placentomes and changes in placentome maturation in ewes showing normal fetal growth despite being at high altitude (Penninga & Longo, 1998). Similar placentome maturational changes are also seen in sheep pregnancies showing hypoxia at sea level (Penninga & Longo, 1998) (Table 3).

Whilst one study reports beneficial adaptions in placental angiogenesis with chronic exposure to 11% oxygen (11% hypoxia) (Cahill et al., 2018), typically 12% hypoxia and lower in rats or mice results in overall decreased placental weight, reduced labyrinthine surface area, a thicker barrier to diffusion and fewer blood spaces in association with severe reductions in fetal weight (Cuffe et al., 2014; Higgins et al., 2015; Kimball et al., 2015) (Table 3). In guinea pigs, while exposure to 10.5% oxygen (10.5% hypoxia) in the last 14 days of pregnancy is linked to increased placental proliferation (indicated by proliferating cell nuclear antigen; PCNA), fetal weight is still reduced (Elsamadicy & Thompson, 2022). In part, the effect of hypoxia appears to be modified by fetal sex (Cuffe et al., 2014; Elsamadicy & Thompson, 2022). For instance, female, but not male, placentas exhibited reduced labyrinth blood spaces with 12% oxygen in mice (Cuffe et al., 2014), and female placentas only showed enhanced PCNA protein abundance in response to 10.5% hypoxia in guinea pigs (Elsamadicy & Thompson, 2022). However, it is also important to note that the effect of hypoxia on placental structure can sometimes be linked to reductions in maternal food intake (Higgins et al., 2015). Finally, in guinea pigs, where the uterine blood flow to the placenta has been constricted from mid‐gestation, labyrinthine vascularization, trophoblast volume and surface area are decreased and track with the poor fetal development observed (Lopez‐Tello et al., 2018) (Table 3).

#### 2.6.2 Changes in placental function

There are also functional adaptations of the placenta in response to hypoxia exposure in humans and animal models (Table 3). Placental glucose transporter activity is reduced although system A or L transporter activity is maintained for women at high altitude compared to sea level (Vaughan et al., 2020; Zamudio et al., 2006). System A transporter activity is also unaffected in the placenta from women with pre‐eclampsia or pregnancy‐induced hypertension (Dicke & Henderson, 1988; Shibata et al., 2008), but taurine transporter activity is compromised (Desforges et al., 2013). In mice, placental glucose transport is adaptively upregulated and system A amino acid transfer unaffected in response to 13% hypoxia in late gestation (Higgins et al., 2015), whereas, glucose supply by the placenta is not upregulated and system A amino acid transport is diminished with expose to 10% oxygen (10% hypoxia) in late gestation (Higgins et al., 2015). The differential effects of 13% versus 10% hypoxia on nutrient transport function are consistent with their varying effects on fetal growth (Higgins et al., 2015). Finally, there are changes in the expression of glucose (*Slc2a1*) and amino acid (*Slc38a1*) transporters in the placenta with 12−13% hypoxia, but not with 10% hypoxia or less (Cuffe et al., 2014; Higgins et al., 2015; Trollmann et al., 2007). Thus, there are adaptive changes in placental nutrient transport function that depend on the severity and type of hypoxic insult and these relate to fetal growth outcomes.

### 2.7 Extremes of maternal age

#### 2.7.1 Changes in placental structure

Extremes of maternal age, namely young or advanced maternal age, can also impact placental physiology. In growing teenage mothers, placental morphology appears to be normal (Hayward et al., 2011), whilst in women of advanced age who deliver low birthweight babies, there are increased syncytial nuclear aggregates and decreased rates of proliferation in the placenta (Lean et al., 2017). In mice, advanced age is associated with overgrowth of the placenta sustaining growth‐restricted viable fetuses, but no change in placental size was observed for the growth‐restricted non‐viable pups in the litter (Lean et al., 2017) (Table 4). In rats, advanced age leads to an expansion of the labyrinthine zone, particularly for female fetuses, although fetuses of both sexes are growth‐compromised (Napso et al., 2019) (Table 4).

#### 2.7.2 Changes in placental function

There is lower system A activity and *Slc38a1* and *Slc38a2* expression in the placenta of teenagers than adults, and the extent of the effect was determined by whether the teenagers were growing or no longer growing (Hayward et al., 2012). In pregnant women who are considered at an advanced age, placental system A and taurine transporter activity are elevated, which may serve to optimize fetal growth (Lean et al., 2017). In direct contrast to findings in women, advanced age in mice leads to decreased placental system A and taurine transporter activity, with the greatest reductions seen for fetuses that were both growth‐restricted and non‐viable (Lean et al., 2017) (Table 4). Finally, although fetuses have reduced growth, there are no overt changes in the expression of glucose (*Slc2a1*, *Slc2a3*) or system A (*Slc38a1*, *Slc38a2*, *Slc38a4*) transporter genes (Napso et al., 2019) (Table 4). Together the data suggest that there are species differences in the adaptive responses of the placenta to extremes of maternal age.

## 3 MECHANISMS MEDIATING PLACENTAL ADAPTIVE RESPONSES IN NORMAL AND ADVERSE ENVIRONMENTS

### 3.1 Imprinted genes

Imprinted genes are a class of genes that are expressed from one of the two inherited parental chromosomes and play highly important roles in regulating normal fetal and placental development (Lim & Ferguson‐Smith, 2010). Insulin‐like growth factor (*Igf2*) was one of the first genes to be identified as imprinted and has been most studied in the context of adaptations in placental physiology during development and with environmental cues in animal models.

In mice, complete ablation of *Igf2* results in placental growth restriction, malformation of the labyrinthine zone (including defective vascularization, increased barrier thickness and reduced surface area), impaired amino acid transport capacity and fetal growth restriction (Baker et al., 1993; Matthews et al., 1999). Deletion of the *Igf2P0* transcript, which is expressed by the labyrinthine zone in mice, also leads to placental growth restriction and compromised labyrinthine zone formation. However, these alterations are compensated by adaptive upregulation of glucose, glutamine, system A amino acid and calcium transport, and as a result, fetal growth is preserved up until close to term (Constancia et al., 2005; Dilworth et al., 2010; McIntyre et al., 2019). Adaptive regulation of nutrient transport in the *Igf2P0* null mutant is mediated by other placental *Igf2* transcripts and fetal IGF2, which act as demand signals helping the placenta to match fetal growth needs during pregnancy (Sferruzzi‐Perri et al., 2017). Recent cell‐specific gene manipulations in mice have indeed revealed that fetus‐derived IGF2 is needed for the appropriate differentiation of trophoblast lineages and expansion of the placental vasculature to support normal fetal growth during normal gestation (Sandovici et al., 2022). In genetically unaltered mouse litters, expression of the *Igf2P0* isoform is greater alongside beneficial structural and nutrient transport changes in the lightest compared to the heaviest placenta supporting normal fetal growth (Coan et al., 2008). However, there is no difference in the placental *Igf2* expression between the lightest and heaviest fetuses within wild‐type normal litters (Salazar‐Petres et al., 2022). Together, these findings highlight that IGF2 plays key roles both in supporting growth and development of the conceptus during gestation and in response to natural and genetically induced mismatches between fetal demand and placental supply of resources.

There are also data suggesting a role of IGF2 in facilitating adaptations in placental phenotype during unfavourable intrauterine environments. In particular, beneficial morphological and functional changes in the placenta that are seen with maternal undernutrition in mice are abolished when the *Igf2P0* isoform has been knocked out, and this results in early, more severe fetal growth stunting (Sferruzzi‐Perri et al., 2011). In mice, there are also changes in *Igf2* (all isoforms and/or *Igf2P0* specifically) that are coincident with beneficial morphological and functional changes in the placenta exposed to diet‐induced obesity (King et al., 2013; Sferruzzi‐Perri et al., 2013; Song et al., 2017) and 13% hypoxia (Higgins et al., 2015). In guinea pigs exposed to late gestational 10% hypoxia, there is also upregulation of *Igf2* by the placenta showing enhanced proliferation and this is dependent on the sex of the fetus as changes are only observed in the females (Elsamadicy & Thompson, 2022). There is also upregulated *Igf2* expression by the placenta of female, but not male, fetuses in aged rats that also had an expanded labyrinthine zone (Napso et al., 2022). Finally, there is decreased expression or no change in *Igf2* when there are detrimental changes in placental phenotype responding to reduced placental perfusion in mice (Habli et al., 2013), undernutrition in guinea pigs (Olausson & Sohlstrom, 2003), as well as with 12% hypoxia in mice, although in the latter, the effect is only seen in female fetuses (Cuffe et al., 2014). Nonetheless, it may not just be down to *Igf2* and the specific insult, and species studied may be important. For instance, other work has found *Igf2* to be stably expressed in the placenta even when there are environmentally mediated changes in placental phenotype with reduced placental perfusion in guinea pigs (Carter et al., 2005), or undernutrition in sheep (McMullen et al., 2005). Further, changes in the placental expression of additional imprinted genes, including *Igf2r*, *H19*, *Dlk1*, *Grb10* and *Slc38a4*, have been reported in different animal models subject to unfavourable gestational environments (Coan et al., 2008, 2011; Cuffe et al., 2014; Lesage et al., 2002; Lin et al., 2012; Sferruzzi‐Perri et al., 2013; Van Gronigen Case et al., 2021). Thus, imprinted genes are important for mediating adaptive responses in placental physiology, in response to both developmental and environmental cues.

### 3.2 Metabolic signalling pathways

Metabolic signalling pathways have been also identified as key candidates involved in mediating changes in placental physiology in response to developmental and environmental cues. The pathways that have received much attention to date are the phosphoinositol‐3 kinase (PI3K), mechanistic target of rapamycin (mTOR), and AMP‐activated protein kinase (AMPK) signalling pathways, which are summarized below. Note that no data are available regarding the relationship between these signalling pathways and adaptive changes in the placenta with extremes of maternal age.

#### 3.2.1 PI3K

The PI3K signalling pathway has been of interest in the control of fetal growth as it is primarily responsible for mediating the metabolic, proliferative and pro‐survival effects of insulin and growth factors, like IGF2. In mice, inactivation of the PI3K isoform, p110α, in the developing conceptus results in malformation of the labyrinthine zone, with reduced fetal vessel density, exchange surface area and a thicker barrier to diffusion (Sferruzzi‐Perri et al., 2016). While fetuses are growth‐restricted in early gestation in this PI3K‐p110α deficient mouse model, the deficit in fetal growth is less towards term as the morphologically impaired placenta adaptively transports more glucose and amino acid (system A) to the fetus (Sferruzzi‐Perri et al., 2016). Cell‐specific gene targeting in mice has since revealed that retention of PI3K‐p110α signalling ability in the trophoblast lineage of the developing conceptus is critical for mediating the adaptive upregulation of placental system A amino acid transport that is optimizing fetal growth towards term (Lopez‐Tello, Khaira, et al., 2019). Other work has indeed highlighted the important role for PI3K signalling in promoting the formation and function of trophoblast lineages (Lee et al., 2019). There is also recent information showing that the sex of the fetus defines the impact of PI3K‐p110α deficiency on murine conceptus growth in vivo, with males more affected than females (Pereira‐Carvalho et al., 2022). Finally, additional work has shown that loss of PI3K‐p110α in the mother, which affects her metabolism and growth during pregnancy (Lopez‐Tello, Salazar‐Petres, et al., 2022) is linked to an enlarged labyrinthine zone yet normal fetal weight because placental glucose transport is downregulated (Sferruzzi‐Perri et al., 2016). In wild‐type mice, PI3K signalling (informed by AKT activation, which is downstream of PI3K) is reduced in the placenta supporting the lightest males in the litter and may explain the decreased capacity for glucose transport when compared to the heaviest males (Salazar‐Petres et al., 2022). No such effects are seen when comparing the lightest and heaviest females. There are also data from different animal species showing changes in the PI3K signalling pathway in the placenta exhibiting adaptive alterations in structure and function with undernutrition (Ma et al., 2011; Sferruzzi‐Perri et al., 2011; Zhu et al., 2007), diet‐induced obesity (Sferruzzi‐Perri et al., 2013; Song et al., 2017) and hypoxia (Elsamadicy & Thompson, 2022; Higgins et al., 2015; Kimball et al., 2015). However, the direction of change in PI3K signalling in the placenta is not always consistent and can be influenced by fetal sex.

#### 3.2.2 mTOR

The mTOR pathway integrates multiple cellular cues related to nutrient availability, energy status and growth factor signalling, which are likely to be altered developmentally and in response to altered gestational conditions. The mTOR pathway regulates various aspects of cell function, but particular key roles are involved in the control of gene expression and protein synthesis. In cell‐based set‐ups using human trophoblast, the activity of glucose and specific amino acid transporters (including system A and L) depended on intact mTOR signalling (Roos et al., 2009; Rosario et al., 2012). In women, there are changes in the expression of genes encoding mTOR signalling components that relate to maternal dietary composition, but these changes are not always correlated with the expression of nutrient transporters, and fetal sex may play a role (Brett et al., 2015, 2016). For instance, mTOR expression is lower in the placenta of males, but not females from obese women (Brett et al., 2016). In women with obesity delivering overgrown babies, there is hyperactivation of the mTOR pathway and elevated amino acid transporter levels in the placenta (Jansson et al., 2013). In undernourished mice, mTOR signalling is downregulated in the placenta at a time when placental, but not fetal, growth is compromised (Sferruzzi‐Perri et al., 2011). In diet‐induced obese mice showing fetal overgrowth, mTOR signalling in the placenta is enhanced in line with greater placental glucose and amino acid delivery to the fetus (Rosario et al., 2016). Placental mTOR signalling is also hyperactivated in the placenta of rats showing enhanced feto‐placental growth in response to a maternal high‐fat diet (Gaccioli et al., 2013). Although there was no change in fetal size in a different model of maternal high‐fat diet in rats, placental mTOR signalling was upregulated in line with elevated glucose (*Slc2a3*) and amino acid transporter (*Slc38a2*) levels, and this effect was only seen for male fetuses (Song et al., 2017). Work in mice has indeed found that mTOR is important for the regulation of glucose transporter (SLC2A3) protein levels in the placenta of high fat diet‐fed mice (Wang et al., 2021). However, in obesity models where fetal size at term is not enhanced or even lower, placental mTOR signalling is reported to be unchanged or reduced and tends to track with reductions in placental size or efficiency (Lager et al., 2014; Ma et al., 2010; Mark et al., 2011; Sferruzzi‐Perri et al., 2013). In the case of exposure to maternal inhalation hypoxia in mice, there is increased activation of the mTOR pathway in the placenta, which appears to align with improvements in placental size and labyrinthine zone morphology even though fetal growth restriction occurs (Matheson et al., 2015), whereas in rats, maternal hypoxia leading to both reduced placental and fetal growth is related to a decrease in mTOR signalling in the placenta (Kimball et al., 2015).

#### 3.2.3 AMPK

The AMPK pathway is activated by depleted cell energy levels and functions to stimulate glucose uptake and lipid oxidation to restore energy levels. Studies in cultured trophoblast have demonstrated the importance of intact AMPK signalling for differentiation, glucose uptake and adaptations in system A amino acid transport capacity (Carey et al., 2014). In mice, recent work has found that the placenta supporting the lightest fetuses in the litter exhibits increased AMPK activation in the labyrinthine zone, which could have implications for the functional and morphological adaptations seen (Salazar‐Petres et al., 2022). In nutrient‐restricted sheep, AMPK signalling is also increased in the placental cotyledon and this relates to adaptive upregulation of nutrient transfer capacity (Ma et al., 2011). In diet‐induced obese mice, AMPK activation in the placenta is reduced and is in accordance with reduced formation of the labyrinthine compartment in male, but not female, fetuses (Napso et al., 2022). In contrast, in over‐nourished pregnant sheep, placental cotyledon AMPK activation is less in line with poor vascular development (Zhu et al., 2009). Finally, data from mice exposed to gestational hypoxia demonstrated that AMPK activation levels in the placenta correlated with alterations in placental morphology, amino acid transport and fetal growth (Higgins et al., 2015; Skeffington et al., 2015).

### 3.3 Subcellular constituents (mitochondria)

Mitochondria are the main cellular manufacturers of energy required for the placenta to grow and transport nutrients to the fetus (Lu & Sferruzzi‐Perri, 2021). They are responsive to environmental cues, and changes in their function have been implicated in placental adaptations during development and with unfavourable gestational environments. In particular, in humans, there are decreases in placental mitochondrial oxidative phosphorylation capacity and metabolic flexibility in women with obesity delivering normal birthweight or large for gestational age babies, but these effects are partly influenced by sex (Mele et al., 2014). How this may relate to the control of mitochondrial biogenesis and electron transfer proteins by mTOR signalling in the placental trophoblast requires study (Rosario et al., 2019).

In mice, there are sex‐specific changes in mitochondrial bioenergetics and regulatory proteins in the placental labyrinthine zone supporting the lightest fetuses in the litter; for example, with females showing lower complex I and males showing lower complex III and V levels (Salazar‐Petres et al., 2022). There is also a sex‐specific increase in mitochondrial reserve capacity of the placental labyrinthine zone in response to PI3K‐p110α signalling deficiency (Pereira‐Carvalho et al., 2022). Whilst mitochondrial respiratory capacity is preserved, there are sex‐dependent differences in the effect of diet‐induced maternal obesity on the abundance of proteins regulating mitochondrial formation and dynamics, for instance with females showing elevated biogenesis marker proliferator‐activated receptor γ coactivator 1‐α (PGC1α) and males instead showing elevated fission protein dynamic‐related protein 1 (DRP1) (Napso et al., 2022). Maternal inhalation hypoxia in mice and guinea pigs is linked to decreased placental capacity for mitochondrial oxidative phosphorylation and serves as a compensatory mechanism to spare oxygen for transfer to the fetus (Matheson et al., 2015; Sferruzzi‐Perri et al., 2019; Song et al., 2019). However, the specific nature of the effect on placental energy metabolism with gestational hypoxia can be sex‐specific, with males, but not females, affected (Song et al., 2019). Mitochondria are inherited from the mother. Work is required to understand the relationship between maternal mitochondrial changes and placental mitochondrial function (Sferruzzi‐Perri, 2021).

### 3.4 Sexomes and fetal signalling

As mentioned in brief in the above sections, sexually dimorphic adaptations in placental structure and function have been reported. Whether these differences relate to males and females having different growth rates in utero, and hence different fetal demand signals for nutrients and oxygen, is unclear (Clifton, 2010; Kalisch‐Smith et al., 2017). The placenta expresses receptors for sex hormones, namely oestrogen and testosterone (Fowden & Forhead, 2015; Salazar‐Petres et al., 2022), and hence the machinery is in place for signals from the fetal gonads to execute responses in the placenta. Finally, the sex of the placenta is the same as the fetus, as it expresses the same complement of sex chromosomes (sexomes). Sexome‐encoding genes play important roles in regulating placental formation and function (Cocchia et al., 2000; Cunningham et al., 2010; Jackman et al., 2012; Jiang et al., 2012; Li & Behringer, 1998; Withington et al., 2006) and could be key candidates in mediating adaptive responses of the placenta to different gestational conditions. However, work specifically exploring sexually divergent placental response has yet to be untaken.

## 4 SUMMARY AND CONCLUSION

**FIGURE 1:**
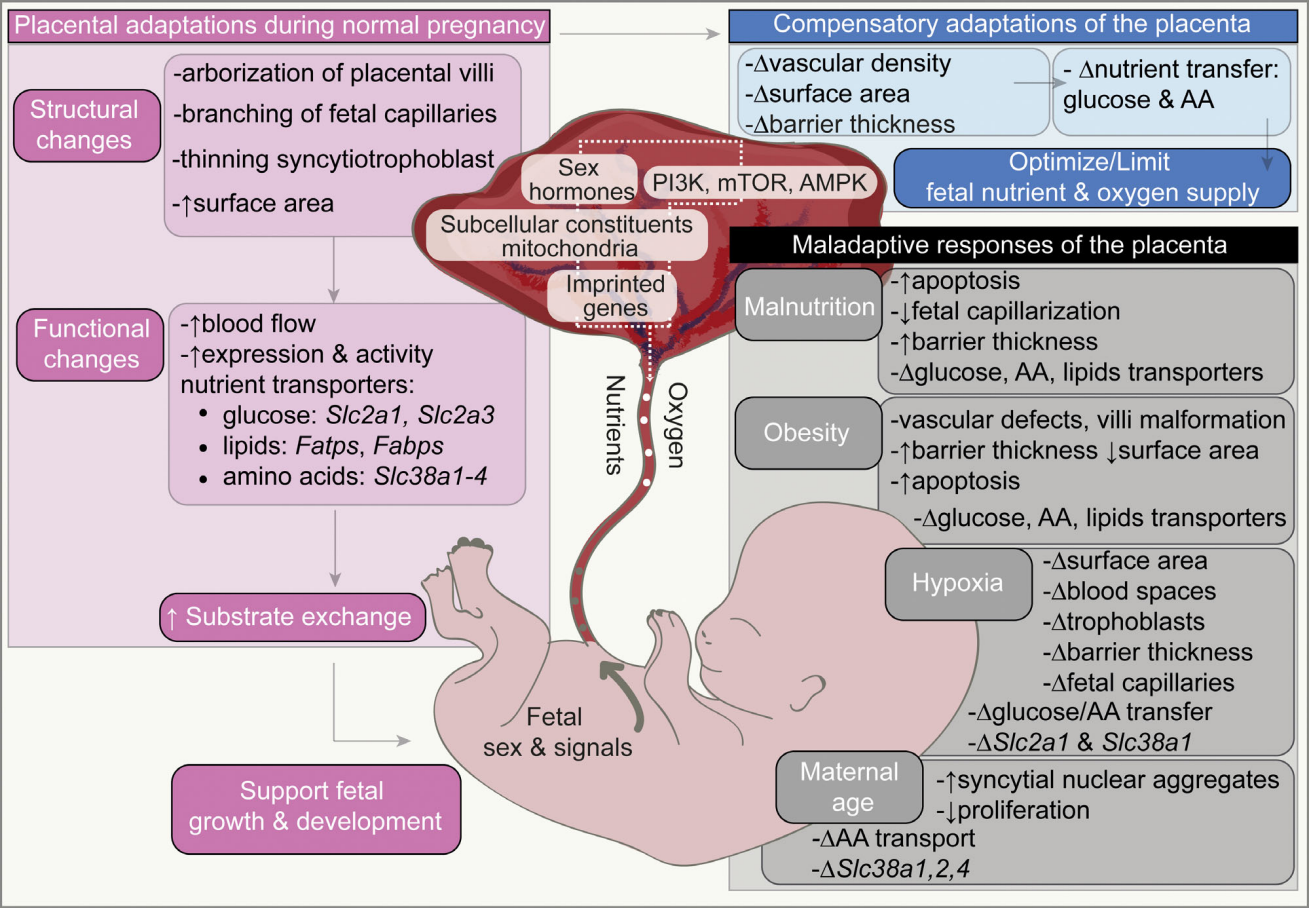
Summary figure illustrating the adaptations in placental structure and function that occur developmentally and in response to different environmental conditions during pregnancy. AA, amino acids; Δ, altered; ↑, increase; ↓, decrease.

To summarize, the placenta has a remarkable ability to adapt its structure and function during development and in response to different stimuli (Figure 1). These changes are facilitated by alterations in imprinted gene expression, signalling pathway activity and mitochondria function, which modulate proliferation, nutrient uptake and metabolism in the placenta. Across the species studied, adaptations in the placenta may have varying effects; they either act to optimize fetal nutrient and oxygen supply and hence fetal growth in the prevailing environment, or instead serve to limit fetal substrate supply, to slow down fetal growth rate in an effort to match substrate provision during gestation. What determines these two opposing placental responses is unclear, but data indicate that they relate to the type, timing, duration and severity of the gestational challenge. There are also differences between species in terms of what placental responses are initiated, how they are executed and what they may mean with regards to fetal growth outcomes. It is highly likely that species differences in the placenta relate to different levels of constraint and competition for resources between the mother and her developing fetus(es) (Fowden & Moore, 2012). Work has also highlighted that there can be important sex differences in placental responses, but few have specifically studied this and furthermore the mechanisms bringing about sexually dimorphic changes are yet to be discovered. Imprinted gene expression and signalling pathway changes are interconnected and likely work together to bring about changes in placental structure and transport function. However, whether changes in imprinted gene expression and signalling pathway are the cause or consequence of the ensuing placental phenotype is unclear (Sferruzzi‐Perri et al., 2011). It is also highly likely that even when similar placental adaptive responses are observed in the same species, genes and signalling events underpinning such adaptations will be distinct (Hayward et al., 2018). The contributions of additional factors, including the by‐products of the maternal microbiome, which is responsive to environmental cues in triggering placental adaptations, are also likely to be highly relevant (Lopez‐Tello, Schofield, et al., 2022). The role of the paternal environment in shaping placental development and function in the context of fetal growth is an area which must be considered in further work (McPherson et al., 2015; Morgan et al., 2021; Pepin et al., 2022). Work using novel platforms like placenta‐on‐a‐chip to study physiological responses (Lee et al., 2016) and mathematical and predictive modelling to understand the changes in the placenta that define fetal growth outcomes (Lewis et al., 2020; Yong et al., 2022) will be key for taking the next steps in the field. Work in this area is fundamentally important for understanding divergent pregnancy outcomes in women and mammalian species more generally, as well as how changes in prenatal development that link to poor postnatal health may occur (Lewis &, Sferruzzi‐Perri, in press; Sferruzzi‐Perri et al., 2009).

## AUTHOR CONTRIBUTIONS

Amanda N. Sferruzzi‐Perri designed the study. Amanda N. Sferruzzi‐Perri, Jorge Lopez‐Tello and Esteban Salazar‐Petres all collected and interpreted the data. Amanda N. Sferruzzi‐Perri prepared the first draft of the manuscript and Jorge Lopez‐Tello and Esteban Salazar‐Petres edited the paper for its content. All authors approved the final version of the manuscript. They also agree to be accountable for all aspects of the work in ensuring that questions related to the accuracy or integrity of any part of the work are appropriately investigated and resolved. All persons designated as authors qualify for authorship, and all those who qualify for authorship are listed.

## CONFLICT OF INTEREST

The authors have no conflicts, including financial interests, to declare.
